# Toxicity and Loss of Mitochondrial Membrane Potential Induced by Alkyl Gallates in *Trypanosoma cruzi*

**DOI:** 10.1155/2015/924670

**Published:** 2015-01-28

**Authors:** Rogério Andréo, Luís Octávio Regasini, Maicon Segalla Petrônio, Bruna Galdorfini Chiari-Andréo, Aline Tansini, Dulce Helena Siqueira Silva, Regina Maria Barretto Cicarelli

**Affiliations:** ^1^Instituto de Química, Universidade Estadual Paulista (UNESP), Rua Professor Franscisco Degni 55, Bairro Quitandinha, CP 355, 14800-900 Araraquara, SP, Brazil; ^2^Faculdade de Ciências Farmacêuticas, Universidade Estadual Paulista (UNESP), Rodovia Araraquara-Jaú, km 1, CP 355, 14801-902 Araraquara, SP, Brazil

## Abstract

American trypanosomiasis or Chagas disease is a debilitating disease representing an important social problem that affects, approximately, 10 million people in the world. The main aggravating factor of this situation is the lack of an effective drug to treat the different stages of this disease. In this context, the search for trypanocidal substances isolated from plants, synthetic or semi synthetic molecules, is an important strategy. Here, the trypanocidal potential of gallates was assayed in epimastigotes forms of *T. cruzi* and also, the interference of these substances on the mitochondrial membrane potential of the parasites was assessed, allowing the study of the mechanism of action of the gallates in the *T. cruzi* organisms. Regarding the preliminary structure-activity relationships, the side chain length of gallates plays crucial role for activity. Nonyl, decyl, undecyl, and dodecyl gallates showed potent antitrypanosomal effect (IC_50_ from 1.46 to 2.90 *μ*M) in contrast with benznidazole (IC_50_ = 34.0 *μ*M). Heptyl gallate showed a strong synergistic activity with benznidazole, reducing by 10^5^-fold the IC_50_ of benznidazole. Loss of mitochondrial membrane potential induced by these esters was revealed. Tetradecyl gallate induced a loss of 53% of the mitochondrial membrane potential, at IC_50_ value.

## 1. Introduction

Chagas disease (or American trypanosomiasis) was recognized by World Health Organization as one of thirteen neglected tropical diseases in the world [1]. It was estimated that over 10 million people are infected, and Latin America was considered the area of the highest prevalence [2]. This parasitic disease is caused by* Trypanosoma cruzi *(family, Trypanosomatidae and order, Kinetoplastida), a hemoflagellate protozoa [3], which can be found on several strains with different mechanisms of pathogenesis, immunogenicity, treatment response, and epidemiology [4]. The development of infection includes an acute phase which lasts up to six months after infection, an indeterminate phase with no symptoms, and a chronic phase in which approximately 30% of patients show clinical evidence of heart disease or megavisceras [5].

The current chemotherapy uses benznidazole, a nitroimidazole derivative, which is orally administered in acute phase and short-term chronic phase [6, 7]. The usefulness of this drug is limited by its narrow therapeutic window and due to serious side effects, such as anorexia, nausea, headache, paresthesia, peripheral neuropathies, dermatitis, central nervous system (CNS) depression, and maniac symptoms [7, 8]. However, several parasite subpopulations with different host tissue's tropism contribute to the low clinical efficacy of this drug [9]. Therefore, there are efforts for the discovery and design of new therapeutic compounds to treat Chagas disease, due to the complexity of this disease.

Gallic acid (3,4,5-trihydroxybenzoic acid) is a precursor of hydrolysable tannins in the biosynthesis that occurs in plants [10] and its natural and semisynthetic derivatives have been associated with a wide variety of biological activities, such as antiproliferative [11–13], chemopreventive [14], antihemolytic [15], antioxidant [16], and anti-inflammatory [17] activities. However, the main interest in gallic acid and in its derivatives has been associated with their antimicrobial properties. It has been shown that* n*-octyl gallate possesses fungicidal activity against* Saccharomyces cerevisiae* and* Zygosaccharomyces bailii* in any stage of their growth [18, 19]. Leal and coworkers reported the potent fungitoxicity of* n*-nonyl gallate against yeasts, dermatophytes, and hialohyphomycetes [20]. Lauryl gallate showed antibacterial activity against Gram-positive bacteria, including methicillin-resistant* Staphylococcus aureus* (MRSA) [21, 22].

Thus, the aim of this study was to investigate the antitrypanosomal activity of gallic acid and its esters against epimastigote forms of* Trypanosoma cruzi*. Further analysis involving interactions generated by combinations between alkyl gallates and benznidazole was also carried out. In addition, the apoptosis-inducing activity by alkyl gallates was studied through the parasite mitochondrial membrane potential assay.

## 2. Material and Methods

### 2.1. Compounds

The compounds tested in this study were synthesized as described previously [14, 15].

### 2.2. Parasites

Epimastigote forms of* Trypanosoma cruzi* of Y strain, described by Silva and Nussenzweig (1953) [23], which is considered a standard strain of type I, were grown in LIT media (liver infusion tryptose media) at 28°C [24].

### 2.3. Cytotoxicity Assay Using MTT

The cytotoxicity assay with epimastigotes* T. cruzi* was performed using the MTT colorimetric method as described by Muelas-Serrano et al. (2000) [25] with modifications described in Cotinguiba et al. (2009) [26]. The parasites were treated with different concentrations of the substances for 72 hours and then, the parasite viability was estimated by measuring the absorbance at 595 nm. The concentration* versus* response curve was constructed to enable the determination of IC_50_. An equation that describes the curve was obtained using Origin 7.0 software. Means and standard deviations were calculated. ANOVA and Tukey's tests were carried out when necessary. Differences in means were treated as significant when *P* < 0.05. Benznidazole was used as positive control (IC_50_ = 34.0 *μ*M).

### 2.4. Mitochondrial Membrane Potential Assay

The influence of the treatments with the gallates in the mitochondrial membrane potential was assessed. The parasites were treated with the substances during 72 hours using, as standard concentration, the IC_50_ previously determined by the MTT assay. The percentage of parasite cells which suffered loss in the mitochondrial membrane potential due to the treatment with the tested compounds was measured by flow cytometry using JC-1 dye (5,5′,6,6′-tetrachloro-1,1′,3,3′-tetraethylbenzimidazolcarbocyanine iodide, BD MitoScreen kit) according to the manufacturer's instruction [27, 28]. A FacsCanto I cytometer was used, and the data were recorded and analyzed on the software of the equipment. Pentamidine was used as positive control, at 58.7 *μ*M for 24-hour treatment.

## 3. Results and Discussion

The antitrypanosomal activity of a homologous series of alkyl gallates, gallic acid, and other analogues was evaluated against epimastigote forms of* Trypanosoma cruzi*. This study was carried out in order to correlate chemical characteristics of the molecules with the observed activity, including the importance of free hydroxyl groups and side chain length.  Table 1 summarizes the IC_50_ values of these compounds.

Gallic acid (**1**) was found to be totally inactive against* T*.* cruzi *(IC_50_ > 100 *μ*M) and its derivatives up to hexyl gallate (**2**–**7**) exhibited similar IC_50_ values (IC_50_ > 100 *μ*M). Heptyl gallate (**8**, IC_50_ = 37.3 ± 0.9) showed weak activity which increased in the case of longer carbon chain derivatives up to undecyl gallate (**12**, IC_50_ = 1.46 ± 0.0). However, the increase in the carbon chains, becoming bigger than undecyl gallate, led to lower activity, as shown for compounds** 13 **and** 14** (Table 1). Such results indicate that the 3,4,5-trihydroxyphenyl moiety appeared to be necessary but not sufficient for antiprotozoal activity. On the other hand, Kubo et al. (2001) [18] verified that the length of the alkyl chain is not a major contributor but plays an important role in eliciting the activity of the gallates.

Among the thirteen tested gallates (**2**–**14**) esterified with linear alcohols from C1 to C14, best results were observed for esters with chain lengths ranging from 9 to 12, which present *C*log⁡⁡*P* values of 4.11−5.30, as reported previously by Rosso et al. (2006) [16].

*n*-Undecyl gallate (**12**), the most active derivative, with IC_50_ of 1.46 *μ*M (*C*log⁡⁡*P* = 4.90), was twenty-three times more potent than benznidazole, used as positive control (IC_50_ = 34.0 *μ*M). Such results indicate that esterification led to appearance of antitrypanosomal activity, suggesting that a free carboxyl group was not crucial for protozoal death. In contrast, esters with *C*log⁡⁡*P* values lower than 3.32 or higher than 0.92 exhibited lower potency than the positive control.

Altogether, there is a clear and positive correlation among IC_50_ values, alkyl chain length and its contribution to the lipophilicity, which are in agreement with previous studies of this homologous series [19, 29]. Furthermore, the trypanocidal potential increased with the increase in the number of carbons found on the side chain until reaching the maximum activity, in this case at* n*-undecyl gallate (**12**), and longer carbon chains showed lower trypanocidal activity, reaching a cutoff at* n*-tetradecyl gallate (**14**). If the homologs longer than** 14** might show antitrypanosomal activity, their IC_50_ values will be superior at 17.6 *μ*M, representing low potency, not corroborating for further chemical or biological investments.

In previous studies, the cutoff phenomenon was observed for alkanols, which was correlated with their amphipathic properties. In the case of alkyl gallates, their amphiphilicity appeared to be dependent on the presence of two features: hydrophilic phenolic hydroxyls and hydrophobic alkyl chain. Fujita and Kubo (2002) [19] reported the antifungal activity of gallates, suggesting that these compounds possessed amphiphilic capacity and surfactant properties and they might act disrupting the fluidic bilayer membrane, leading to fungal death.

The synergistic effects of the combination using gallic acid and its esters with benznidazole, the antichagasic drug, were also evaluated. The definitions and mathematical determination of the formula for synergism were used as described by Hellmann et al. (2010) [30]. Briefly, the fractional inhibitory concentration (FIC) index was calculated by using the formula FIC index = *A*/*B* + *A*/*C*. In this formula, *A*, *B*, and *C* were IC_50_ of gallate with benznidazole, IC_50_ of gallate alone, and IC_50_ of benznidazole alone, respectively. From this calculation, a FIC index ≤ 0.5 was considered evidence of synergistic effect, as this value can be generated only if the concentrations of both compounds in combination do not exceed one-quarter of the IC_50_ of either drug tested alone. A FIC index > 4.0 indicates antagonism. When tested alone, some gallates (**2**–**7**) have not led to a significant number of epimastigotes toxicity, even at the highest concentration tested (100 *μ*M). Therefore, to calculated FIC index for these compounds, the IC_50_ was arbitrarily defined as 50 *μ*M (Table 2).

The interactions between compounds** 1**–**14 **and benznidazole were highly variable, indicating dependence on the gallates chain length. Association between gallic acid (**1**) and short esters (**2**–**4**) with benznidazole was indifferent. Additive interaction was observed for inactive short esters (**5** and** 6**) and most potent long esters, which exhibited FIC index values ranging from 0.560 to 0.765. Interestingly, medium chain length gallates (**7**–**9**) and the long chain length ester** 14** with FIC index values bellow 0.257 exhibited synergism. The most significant synergistic effect was observed for heptyl gallate (**8**, FIC = 0.084), which reduced by 10^5^-fold the concentration of benznidazole necessary to inhibit cell growth in 50% (IC_50_ of 37.3 ± 0.93 *μ*M).

Synergism was observed at *μ*M level, evidencing a strong antitrypanosomal activity and the potential of medium and long chain alkyl gallates as prototypes for further studies and development of novel therapeutic agents. It is also worth mentioning that the significant results from the association of benznidazole and alkyl gallates, which enabled the use of smaller doses of benznidazole, extended dosing intervals between consecutive administrations and short-term treatment leading to fewer and less intense side effects.

Few reports have been found on the antimicrobial synergistic effect against trypanosomatidae protozoa. Urbina and coauthors (1988, 1995, 2002, 2009) [31–34] described systematic studies involving antifungal drugs and their associations as trypanocidal combined agents for treatment of Chagas disease, such as azoles (ketoconazole and posaconazole) with mevinolin, terbinafine, aspirin, and amiodarone. Recently, the interaction between benznidazole and parthenolide was investigated against* T. cruzi*, which evidenced synergistic and additive activities against epimastigotes and trypomastigotes, respectively.

**Figure 1:**
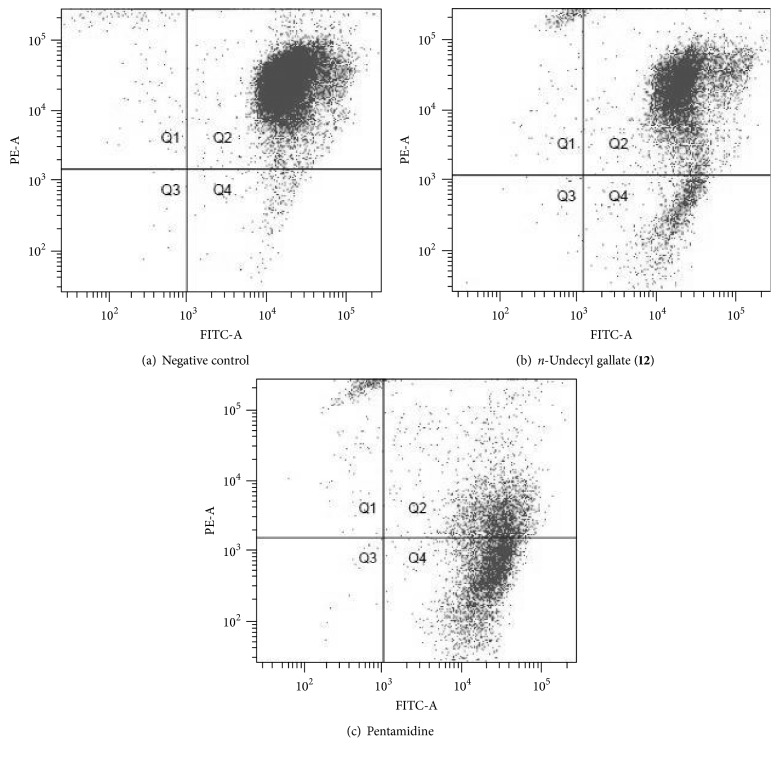
Flow cytometry analysis of epimastigotes forms of* T. cruzi* Y strain, without any treatment (a), treated with 1.46 *μ*M of* n*-Undecyl gallate (**12**) for 72 h (b), and treated with 58.7 *μ*M of pentamidine for 24 h (c).

Additionally, trypanocidal gallates (**8**–**14**) were investigated for their influence on the mitochondrial membrane potential. Considering that the loss of mitochondrial membrane potential is a typical characteristic of apoptotic cells [35], it was used to elucidate a possible mode of parasite death. In living cells, JC-1 dye crosses the plasmatic membrane as monomers, penetrating into the mitochondria. This process is controlled by the membrane potential of this organelle. The membrane of healthy mitochondria is polarized and JC-1 is rapidly internalized, increasing the concentration gradient, which may lead to its aggregation. In the flow cytometer, the JC-1 monomers were detected in the FL-1 channel (FITCS), while its aggregates were observed in the red channel FL-2 (PE). On the other hand, injured mitochondria exhibits depolarized membrane and JC-1 remains in the cytoplasm as monomers. Then, we suggest that the cells which loss their mitochondrial membrane potential, which could be apoptotic cells, lose their fluorescence and could be captured in Q2 quadrant, whereas normal cells are detected in Q4 quadrant.  Figure 1 presents the flow cytometry graphs.

Figure 1 presents the flow cytometry analysis of* n*-undecyl gallate (**12**) on* T. cruzi*.

Among the tested gallates, compounds** 8**,** 9**, and** 14** promoted important loss in the mitochondrial membrane potential, which maybe an apoptosis-inducing activity, with percentage of cells with reduction of mitochondrial membrane potential of 46.6, 43.6, and 53.2, respectively. The results are presented on Table 3.

Figure 1(a) shows that untreated epimastigotes (control) did not lose their mitochondrial membrane potential which can be observed due to the high fluorescence detected in FL-2 channel (PE). Similarly, it is observed that* n*-undecyl gallate (**12**) (Figure 1(b)) did not stimulate this influence in the treated cells since it did not induce a reduction of fluorescence as shown by the control, suggesting the maintenance of the membrane potential. In contrast, the cells treated with pentamidine (Figure 1(c)) show intense decrease in the fluorescence represented by PE, suggesting a loss of mitochondrial membrane potential which may indicate apoptosis of the parasites analyzed. The results of flow cytometry for* n*-undecyl gallate (**12**) are presented on Table 3.

The results showed that the gallate esters used in this study induced loss of the mitochondrial membrane potential and maybe apoptosis in the parasites. This indicates that one of the mechanisms of cell death induced by the gallates activity could be similar to the apoptosis. This can be visualized by comparing the results with pentamidine, an apoptosis-inducing drug [36], which led to different percentages of mitochondrial membrane potential relatively to the control. The treatment with some gallates, such as the substances** 8**,** 9**, and** 14**, showed a percentage of loss of mitochondrial membrane potential similar to the pentamidine treatment.

Pentamidine is a dicationic drug which has been used in the last 50 years for the treatment of African trypanosomiasis and antimony-resistant leishmaniasis and is known to cause changes in mitochondrial metabolism [37]. The drug that can cause a collapse in mitochondrial membrane potential leads to an imbalance in intracellular Ca^2+^ [38]. Electronic microscopy transmission studies with cells treated with pentamidine showed condensation and disruption of kinetoplast DNA core and collapse of mitochondrial membrane [39, 40].

Based on the results obtained by the flow cytometry and cytotoxicity analysis, it is possible to infer that other mechanisms of action, different from apoptosis, might be associated with the death of the parasites, since the substances that caused more significant dysfunction in the mitochondrial membrane potential (**8**,** 9**, and** 14**) are not the same substances that according to the cytotoxicity assay cause higher rates of parasites death (**10**,** 11**,** 12**, and** 13**). In addition, maintenance of cell viability might be associated with low affinity or steric hindrance of the molecular target due to its physical-chemical properties.

Another possible mechanism of action of the gallic acid esters would be similar to the results found by Abe et al. (2000) [41]. These authors showed that some gallates are potent inhibitors of the enzyme squalene epoxidase, which is involved in the biosynthesis of ergosterol [42] and, therefore, is essential for the survival of the microorganism.

Ergosterol is essential to the parasite because it is the most abundant sterol found in the membrane of lower eukaryotes and is therefore found in fungi and parasitic protozoa such as* Leishmania* and* Trypanosoma *[43–46].

The inhibition of sterol biosynthesis is associated with the loss of cell membrane fluidity, which might lead to the parasite death through the same mechanism of action of commercial drugs, such as terbinafine [42].

Mitochondria are one of the first organelles to be affected after treatment of* T. cruzi* with ergosterol biosynthesis inhibitors, and even stronger effect may occur when an inhibitor of squalene epoxidase is used [36–47].

Due to the results of our experiments that demonstrated that there are changes in the mitochondrial membrane potential of the parasites when treated with gallates, we suggest that these compounds cause the parasite death by more than one pathway, which probably includes loss of mitochondrial membrane potential, correlated with the induction of apoptosis and, also, interfering in the biosynthesis of ergosterol in the parasites.

The phenol moiety is often associated with cytotoxic effects due to the possible free radical or reactive oxygen species formation, which might be deleterious to the parasite metabolism.

## 4. Conclusions

This study demonstrated that gallic acid is not very toxic to* T. cruzi* epimastigotes. However its esterification yielding gallate esters with different side chain lengths afforded compounds with strong trypanocidal potential, which is probably also due to a change in the mitochondrial membrane potential and an interference in the biosynthesis of ergosterol in the parasites [48]. It is important to clarify that previous studies demonstrate a correlation between epimastigote and trypomastigote forms of* T. cruzi,* the last one the infective form. Thus, this research encourages the development of new research aiming to apply the gallates as an alternative to current therapy against Chagas disease.

## Figures and Tables

**Table 1:** Antitrypanosomal activity of gallic acid (**1**) and *n*-alkyl gallates (**2**–**14**) against epimastigote forms of *Trypanosoma cruzi*. IC_50_ values were expressed in *μ*M.

Compound	R	*C*log⁡*P* ^a^	IC_50_
Gallic acid (**1**)	H	0.89	>100^b^
Methyl gallate (**2**)	CH_3_	0.92	>100^b^
Ethyl gallate (**3**)	CH_2_CH_3_	1.27	>100^b^
Propyl gallate (**4**)	(CH_2_)_2_CH_3_	1.73	>100^b^
Butyl gallate (**5**)	(CH_2_)_3_CH_3_	2.13	>100^b^
Pentyl gallate (**6**)	(CH_2_)_4_CH_3_	2.53	>100^b^
Hexyl gallate (**7**)	(CH_2_)_5_CH_3_	2.92	>100^b^
Heptyl gallate (**8**)	(CH_2_)_6_CH_3_	3.32	37.3 ± 0.9
Octyl gallate (**9**)	(CH_2_)_7_CH_3_	3.72	23.0 ± 5.3
Nonyl gallate (**10**)	(CH_2_)_8_CH_3_	4.11	2.90 ± 0.1
Decyl gallate (**11**)	(CH_2_)_9_CH_3_	4.51	1.50 ± 0.3
Undecyl gallate (**12**)	(CH_2_)_10_CH_3_	4.90	1.46 ± 0.0
Dodecyl gallate (**13**)	(CH_2_)_11_CH_3_	5.30	2.13 ± 0.2
Tetradecyl gallate (**14**)	(CH_2_)_13_CH_3_	6.09	17.6 ± 1.8
Benznidazole^c^	—	—	34.0

^a^Values described by Rosso and coworkers (2006) [16].

^b^Percentage of inhibition at 100 *μ*M.

^c^Positive control.

**Table 2:** Synergism evaluation between hypothetical IC_50_ values used for calculation of FIC index.

Compound	IC_50_ (gallates alone)	IC_50_ (gallates with benznidazole)	FIC index	Interaction type^*^
Gallic acid (**01**)	294^a^	17.4	2.017	I
Methyl gallate (**02**)	271^a^	15.3	1.786	I
Ethyl gallate (**03**)	252^a^	10.2	1.193	I
Propyl gallate (**04**)	235^a^	8.90	1.043	I
Butyl gallate (**05**)	221^a^	7.47	0.878	A
Pentyl gallate (**06**)	208^a^	6.02	0.708	A
Hexyl gallate (**07**)	196^a^	2.28	0.257	S
Heptyl gallate (**08**)	37.3	0.64	0.084	S
Octyl gallate (**09**)	6.49	0.62	0.165	S
Nonyl gallate (**10**)	0.86	0.44	0.560	A
Decyl gallate (**11**)	0.46	0.24	0.548	A
Undecyl gallate (**12**)	0.47	0.32	0.716	A
Dodecyl gallate (**13**)	0.72	0.51	0.765	A
Tetradecyl gallate (**14**)	6.44	0.58	0.155	S
Benznidazole^b^	8.84	—	—	—

^a^Hypothetical IC_50_ value used for calculating of FIC index.

^b^Positive control.

^*^I, A, and S mean indifferent interaction, antagonism, and synergism, respectively.

**Table 3:** Loss of mitochondrial membrane potential (%) of parasite cells.

Compound	Negative control	Parasite treated with gallates	Positive control
Heptyl gallate (**08**)	1.0	46.6	56.9
Octyl gallate (**09**)	1.0	43.6	56.9
Nonyl gallate (**10**)	1.0	5.9	56.9
Decyl gallate (**11**)	1.0	9.0	56.9
Undecyl gallate (**12**)	1.0	12.5	56.9
Dodecyl gallate (**13**)	1.0	13.4	56.9
Tetradecyl gallate (**14**)	1.0	53.2	56.9
